# The Genetic Jigsaw of Endometrial Polyps

**DOI:** 10.3390/ijms27135655

**Published:** 2026-06-23

**Authors:** Dimitra Lampropoulou, Michail Kalinderis, Liana Fidani, Theodora Katopodi, Maria Chatzidimitriou, Kallirhoe Kalinderi

**Affiliations:** 1 School of Medicine, Faculty of Health Sciences, Aristotle University of Thessaloniki, 54124 Thessaloniki, Greece; dimitralampropoulou2006@gmail.com; 2 Department of Obstetrics and Gynecology, St George’s University Hospital NHS Trust, Blackshaw Road, Tooting, London SW17 0QT, UK; m.kalinderis@hotmail.com; 3 Laboratory of Medical Biology-Genetics, School of Medicine, Faculty of Health Sciences, Aristotle University of Thessaloniki, 54124 Thessaloniki, Greece; sfidani@auth.gr (L.F.); katopodi@auth.gr (T.K.); 4 Department of Biomedical Sciences, Faculty of Health Sciences, International Hellenic University, 57400 Thessaloniki, Greece; mchatzid952@gmail.com

**Keywords:** endometrial polyps, chromosomal rearrangements, mutation, gene expression, endometrial receptivity, infertility

## Abstract

Endometrial polyps are common benign lesions of the uterine cavity characterized by localized overgrowth of endometrial glands, stroma, and vasculature. They are mostly asymptomatic, although in some cases they cause abnormal uterine bleeding and infertility. Increasing evidence indicates that endometrial polyps represent genetically heterogeneous lesions with a multifactorial molecular basis. This review aims to analyze current knowledge on the genetic background of endometrial polyps. For this narrative review article, we searched PubMed and Scopus databases for peer-reviewed research, review articles, and meta-analyses regarding the role of genetics in endometrial polyps, published in the English language with no time restrictions. References of the selected articles for possible additional articles were also screened in order to include most of the key recent evidence. This review highlights the multifactorial genetic landscape underlying the development of endometrial polyps. Current data suggest that these lesions cannot be explained by a single pathogenic mechanism, but rather arise through the interaction of chromosomal changes, somatic and germline genetic variants and dysregulated gene expression. Understanding and integrating these genetic and molecular alterations may improve future diagnostic evaluation, risk stratification, and clinical management of endometrial polyps, although most findings are not yet ready for routine clinical application.

## 1. Introduction

Endometrial polyps are abnormal benign growths composed of endometrial glands, stroma and blood vessels that project from the uterine lining into the uterine cavity [1]. They can occur across all age groups but are most commonly diagnosed in women between 40 and 49 years of age and are observed in both reproductive and postmenopausal periods [1]. Although the majority of endometrial polyps are benign and often asymptomatic, they are involved in a significant number of abnormal uterine bleeding cases [1]. Their clinical importance in reproductive medicine is highlighted because of their association with infertility [1].

Endometrial polyps have been consistently associated with reduced fertility, mainly through impaired embryo implantation [2]. Polyps may mechanically interfere with embryo attachment, changing local endometrial architecture or modifying the molecular environment required for endometrial receptivity, which disrupts implantation. Clinical data support an association between the presence of endometrial polyps and implantation failure, suggesting that these lesions may interfere with early pregnancy establishment through both structural and molecular mechanisms [3].

The exact etiology of endometrial polyps remains poorly understood and their development is considered multifactorial [1]. Several risk factors have been proposed to contribute to polyp formation, including estrogen exposure, altered progesterone responsiveness, inhibition of apoptosis, localized chronic inflammation, and abnormal angiogenesis within the endometrium [1]. The complexity of endometrial polyps’ pathology suggests that they represent heterogeneous entities characterized by localized endometrial dysregulation [1].

Despite the high frequency and clinical importance of endometrial polyps, the molecular mechanisms that impact their development have not been elucidated. However, recent data indicate that genetic and molecular alterations contribute to the pathogenesis of endometrial polyps [1]. This review aims to summarize current knowledge on chromosomal alterations, gene mutations and changes in gene expression in endometrial polyps, providing an integrated framework for understanding their pathogenesis and reproductive implications (Table 1).

## 2. Chromosomal Alterations in Endometrial Polyps

Multiple chromosomal abnormalities, particularly involving regions such as 6p21, 12q13–15, 7q22, and 14q24, have been associated with the development of endometrial polyps. Several cytogenetic aberrations in endometrial polyps have been reported suggesting the presence of distinct genetic pathways underlying their development. In a cytogenetic analysis of 33 histologically benign endometrial polyps, Dal Cin et al. first demonstrated that chromosomal abnormalities are present in the majority of cases and can be used to delineate distinct genetic subgroups. Clonal structural aberrations were identified in 57% of lesions, allowing the authors to classify four major cytogenetic categories: polyps with rearrangements involving 6p21–22 (Subgroup I—the largest subgroup 12/33), those with abnormalities affecting 12q13–15 (Subgroup II—5/33), a smaller group with alterations of 7q22 (Subgroup III—2/33), and a subgroup with normal karyotypes (Subgroup IV—14/33) [4]. It is highlighted that endometrial polyps, although clinically and histologically similar, encompass genetically heterogeneous entities [4].

### 2.1. Chromosome 6p21

Chromosomal rearrangements involving the short arm of chromosome 6 have been identified in benign endometrial polyps, defining 6p21 as a recurrent breakpoint region implicated in their pathogenesis [5]. Evidence from cytogenetic studies suggests that this same terminal 6p cluster (bands p21–p23–pter) is also repeatedly altered in other benign mesenchymal tumors, such as lipomas, uterine leiomyomas and angioleiomyomas, indicating that the region may harbor gene(s) involved in cellular proliferation rather than malignant transformation [19,20]. Moreover, the recurrent involvement of 6p21 in endometrial polyps raises the possibility that this region contains a gene critical for polyp initiation and/or progression, although it remains unclear whether such a gene would act as an oncogene, an oncogene-activator or a tumor suppressor [5]. Candidate genes mapped to this region have biological functions that include stromal proliferation and cytokine signaling which suggest plausible mechanisms through which alterations at 6p21 could contribute to polyp development [5]. The identification of a clonal inv(6)(p21q22) as the sole cytogenetic abnormality in a benign endometrial polyp provides further evidence that supports the non-random involvement of chromosome band 6p21 in endometrial polyp pathogenesis [21]. The specificity of the 6p21 alteration to the polyp was supported by the absence of chromosomal abnormalities in a concurrent uterine leiomyoma from the same patient. Histopathological examination revealed a benign polyp with prominent stromal features, which further supports the fact that 6p21 rearrangements arise within the mesenchymal compartment [21]. The results strengthen the conclusion that disruption of genes located at the 6p21 locus contributes to benign stromal proliferation and defines a recurrent cytogenetic mechanism in a subset of endometrial polyps [21]. The rearrangements in 6p21–22 result in upregulation of high mobility group AT-hook (HMGA1) [6]. *HMGA1* encodes a small non-histone chromatin-binding protein that functions as an architectural transcription factor regulating chromatin structure and gene transcription [22]. *HMGA1* expression is normally low, whereas its upregulation has been associated with abnormal cellular proliferation and tumor-related processes [6]. Rearrangements involving *HMGA1*, which is located at chromosome 6p21, are among the most common structural alterations affecting endometrial polyps and they frequently occur in the stromal component of the polyps [6]. It is suggested that *HMGA1* is accompanied by upregulation of downstream genes such as *PLAG1*, which are known to promote cellular proliferation [6]. These results indicate that *HMGA1* plays a significant role in the molecular pathogenesis of endometrial polyps. From a biological perspective, *HMGA1* alterations are most relevant to the stromal component of endometrial polyps. Rather than indicating malignant transformation, these rearrangements appear to promote benign stromal proliferation and clonal expansion, supporting the concept that at least a subset of endometrial polyps behaves as a benign mesenchymal/stromal neoplasm.

### 2.2. Chromosome 12q13–15

Aberrations in chromosome 12 have also been observed in cases with endometrial polyps. Walter et al. report an example of a benign endometrial polyp exhibiting a structural abnormality of this chromosome. In fact, a clonal inversion, inv(12)(p11.2q13), was detected in approximately one-third of analyzed metaphases, representing the sole karyotypic change identified [23]. The breakpoint at 12q13 lies within a chromosomal region recurrently altered across a spectrum of benign mesenchymal neoplasms—including uterine leiomyomas, lipomas, and pleomorphic salivary gland adenomas—supporting the view that this locus harbors proliferation-associated genes rather than genetic drivers of malignant transformation [23]. Vanni et al. [24] subsequently broadened this observation by reporting two further cases of benign endometrial polyps harboring clonal chromosomal abnormalities that disrupt the 12q13–q15 region: one displaying a t(12;13)(q14–15;q34) and the other an inv(12)(p12–13q14–15). In both cases, the rearrangements were present in a significant subset of metaphases and constituted the sole consistent cytogenetic change, mirroring the isolated 12q inversion previously described by Walter et al. [23]. Together, these findings underscore the recurrent and selective targeting of the 12q13–q15 interval in endometrial polyps and provide compelling evidence that lesions with such alterations represent a distinct cytogenetic subgroup within this otherwise histologically uniform benign tumor type [24]. This observation further reinforces the concept that, despite their benign histology, endometrial polyps can arise through shared cytogenetic pathways, analogous to those present in other non-epithelial soft tissue tumors [23].

Notably, within the region of 12q13–15 lies high mobility group AT-hook 2 (*HMGA2*), also referred to as *HMGI-C* [7]. *HMGA2* encodes a small, chromatin-associated protein distinguished by its rapid migration in polyacrylamide gels. Functionally, it serves as an architectural transcription factor: it binds directly to AT-rich regions of DNA, reshaping local chromatin structure and influencing the expression of nearby genes [25]. *HMGA2* is described as a member of the high-mobility-group I family of nuclear proteins that participate in assembling higher-order transcriptional complexes and whose DNA-binding ability is regulated during the cell cycle [7]. In the study by Bol et al. a complex chromosomal rearrangement involving chromosomes 2 and 12 was identified in an endometrial polyp. The karyotype included inversion, insertion, translocation, and deletions affecting chromosome 12q13–15. Using G-banding and FISH, the breakpoint on chromosome 12 was mapped specifically to the third intron of *HMGA2* [7]. Therefore, some endometrial polyps may form because of rearrangements of *HMGA2*. The fact that the same gene is known to be altered in several other benign tumors, such as fibroids, suggest that endometrial polyps might share molecular mechanisms with these tumors [7]. In a different study, a novel mechanism of *HMGA2* dysregulation in a benign endometrial polyp through high-level gene expression is described [8]. It is mentioned that in some cases aberrant activation of *HMGA2* in benign mesenchymal tumors may arise through amplification of the gene on double minute chromosomes [8].

Double-minute chromosomes are cytogenetic markers of extrachromosomal DNA amplification and may contribute to increased *HMGA2* copy number and expression in a subset of benign endometrial polyps [8,26]. In the study by Dal Cin et al., numerous double-minute chromosomes were identified in all metaphase spreads from the lesion, and it was demonstrated by fluorescence in situ hybridization that these extrachromosomal elements contained multiple intact copies of the full *HMGA2* locus, including both its 5′ and 3′ flanking regions. Immunohistochemistry was shown to reveal strong nuclear expression of HMGA2 protein within the stromal component of the polyp, despite the absence of classical clonal structural abnormalities [8]. Taken together, these findings are considered to expand the recognized spectrum of *HMGA2* alterations in benign endometrial polyps, indicating that amplification through double-minute chromosome formation—rather than chromosomal translocation or inversion alone—can also result in aberrant *HMGA2* expression and may represent an additional, previously undocumented mechanism in the development of these lesions [8].

Another study analyzed genomic and cytogenetic alterations in endometrial polyps arising in postmenopausal breast cancer patients treated with tamoxifen and compared the results with endometrial polyps and normal endometrium from women not exposed to tamoxifen [27]. Chromosomal rearrangements were identified involving 6p21 and 12q13–15 confirming alterations of the *HMGA1* and *HMGA2* loci, although these rearrangements were indistinguishable in type and frequency between tamoxifen-associated and sporadic polyps. In addition, endometrial carcinomas from tamoxifen-treated women did not show these *HMGA*-associated rearrangements, which displayed complex or normal karyotypes [27]. Cryptic inversions affecting 12q15 were detected in some polyps, which further suggests that rearrangements in *HMGA1* and *HMGA2* represent early events in benign polyp development [27]. These findings support that tamoxifen does not induce novel chromosomal alterations in the endometrium but rather promotes polyp formation through pathways already implicated in benign mesenchymal proliferation [27]. Notably, high-depth sequencing studies of benign endometrial polyps have not identified *HMGA1* (*HMGIY*) or *HMGA2* (*HMGI-C*) rearrangements or other large scale genomic alterations, indicating that large scale chromosomal abnormalities are not a universal feature of these lesions [10]. In response to these findings by Sahoo et al. [10], it has been argued that the absence of detected *HMGA1*/*HMGA2* rearrangements in sequencing-based studies likely reflects methodological limitations, as balanced or extragenic breakpoints—previously validated by FISH—are not reliably captured by exon sequencing and may preferentially occur in the stromal component of endometrial polyps [3].

Taken together, the available cytogenetic and FISH-based evidence suggests that *HMGA2* dysregulation represents one of the best-supported stromal mechanisms in endometrial polyp development. Its biological relevance lies in the promotion of benign stromal overgrowth through altered chromatin organization and proliferation-related transcriptional programs. However, because these findings are largely derived from cytogenetic or targeted studies, their prevalence and clinical significance require confirmation in larger compartment-resolved genomic cohorts.

### 2.3. Chromosome 7q22

Alterations involving the chromosomal region 7q22 represent a relatively rare cytogenetic subgroup in endometrial polyps [4]. Although rearrangements affecting 7q22 have been reported in a small subset of cases, the specific genes or molecular mechanisms underlying these alterations have not been clearly defined and the biological significance of this region remains largely unknown. Therefore, 7q22 should currently be regarded as a preliminary cytogenetic finding rather than an established driver of endometrial polyp formation. Its inclusion is important for completeness, but the lack of recurrently identified target genes limits conclusions regarding its direct contribution to polyp biology.

### 2.4. Chromosome 14q24

A further cytogenetic mechanism underlying endometrial polyps is highlighted by the recurrent detection of a t(6;14)(p21;q24) translocation, identified as the sole clonal chromosomal alteration in three independent stromal-predominant lesions [9]. This translocation consistently pairs the 6p21 breakpoint with chromosome 14q24, marking the first report of chromosome 14 involvement in endometrial polyps and defining a previously unrecognized cytogenetic subgroup [9].

In a recent comprehensive genomic profiling study, Reinikka et al. reported that most examined endometrial polyps harbored balanced chromosomal rearrangements, supporting their relationship with a benign neoplastic process [6]. High frequency of *HMGA1* (6p21) and *HMGA2* (12q14–15) rearrangements was observed, with partner breakpoints mapping to regions such as 7p15.2, 10q22 and 14q24, extending earlier cytogenetic observations to a genome-wide level. Benign lesions were characterized by low genomic instability, as the copy-number changes were minimal [6]. These findings support the classification of a subset of endometrial polyps as stromal neoplasms driven primarily by structural genomic alterations rather than aneuploidy [6].

Although 14q24 involvement expands the spectrum of structural alterations in endometrial polyps, its biological interpretation remains less certain than that of *HMGA1*/*HMGA2* rearrangements. At present, 14q24 is best interpreted as a recurrent partner region within balanced rearrangements rather than as an independently validated molecular driver.

## 3. Gene Mutations in Endometrial Polyps

Accumulating evidence indicates that both germline and somatic gene mutations contribute to the genetic landscape of endometrial polyps and may influence their development, susceptibility and recurrence.

A large genome-wide association study analyzing 36,984 women with female genital tract polyps and 420,993 controls from FinnGen, the Estonian Biobank, and the Pan-UK Biobank identified sixteen independent genomic loci significantly associated with polyp formation, thereby providing the first comprehensive evaluation of germline variants predisposing to endometrial polyp development [12]. Across these loci, functional variant-to-gene (V2G) prioritization highlighted several genes with known roles in DNA replication and repair, cell cycle regulation, and proliferative signaling, suggesting that inherited susceptibility to endometrial polyps is mediated through pathways governing genomic stability and controlled cellular proliferation [12]. For example, genes such as Outer Dense Fiber of Sperm Tails 3 (*ODF3*), 26S Proteasome Non-ATPase Regulatory Subunit 13 (*PSMD13*), a catalytic subunit of DNA polymerase δ [13] (*POL3*), leucine-rich repeat 34 (*LRRC34*), Myoneyrin Gene (*MYNN*), Exonuclease 1 (*EXO1*) and Checkpoint Kinase 2 (*CHEK2*) play a significant role in processes related to DNA damage repair which often leads to tumorigenesis [12]. Other genes such as DNA primase, Polypeptide 1, 49 kDa (*PRIM1*), part of a large family of 13 proteins in Tetrahymena [28] (*SFR1*), Phospholipase C Epsilon) (*PLCE1*), Zinc Finger and BTB Domain Containing 38 (*ZBTB38*) and Nuclear Factor I A (*NFIA*) are related to cellular proliferation, a process that characterizes benign neoplasms [12]. Endometrial polyps are also associated with genes that are related to uterine fibroids and endometriosis such as Eukaryotic Elongation Factor, Selenocysteine-Specific (*EEFSEC*), *LRRC34* and *MYNN*. Several identified loci overlapped with regions previously implicated in various malignancies, including endometrial and ovarian cancer, as well as soft-tissue neoplasms, indicating shared genetic architecture between benign polyp growth and tumorigenic processes [12]. Genetic correlation analyses further demonstrated significant associations with increased Body Mass Index (BMI), uterine fibroids, irregular or heavy menses, and reduced Sex Hormone-Binding Globulin (SHBG) levels, implying that hormonal responsiveness and metabolic regulation interact with the identified risk alleles to influence polyp formation [12]. Collectively, these findings support that endometrial polyps possess a distinct germline mutational susceptibility profile enriched for variants affecting DNA damage repair and proliferative pathways, supporting a broader model in which benign overgrowth of endometrial stroma arises from inherited perturbations in cell cycle homeostasis [12]. These germline findings should be interpreted as susceptibility signals rather than direct causal drivers of individual polyp formation. Their main biological relevance is that they link endometrial polyps to inherited variation in pathways regulating DNA repair, cell-cycle control, hormonal responsiveness, and benign tissue overgrowth. However, functional validation is still needed before these loci can be used for risk prediction or clinical stratification.

Somatic mutation analysis indicated a high frequency of oncogenic alterations in endometrial polyps, with *RAS* genes being the most commonly affected. *KRAS* mutations were identified in two of four initially analyzed polyps by whole-exome sequencing [11]. Also, it was demonstrated that 45.7% of endometrial polyps harbored *RAS* mutations, mainly involving *KRAS*, and less frequently *NRAS*, which is unexpectedly high for a benign condition. These mutations were restricted to exon 2 and included well-characterized activating substitutions [11]. Importantly, patients with *RAS* mutations had significantly higher number of polyps compared to mutation-negative patients. In addition, it was revealed that identical *RAS* mutations were present in both glandular and stromal components, which suggests a clonal origin of these lesions [11]. These findings suggest that *RAS* mutations are relatively common in benign endometrial polyps and may contribute to epithelial proliferation and the development of multiple lesions, despite the absence of malignant progression [11]. The biological significance of *RAS* mutations in endometrial polyps should therefore be interpreted cautiously. Although these variants may promote epithelial proliferation and may contribute to the development of multiple lesions, their presence in a benign histological context does not necessarily imply malignant potential. Instead, *RAS* mutations may represent compartment-specific epithelial events that coexist with stromal alterations and contribute to local polyp growth or persistence.

A subset of endometrial polyps was found to harbor somatic mutations in *UBE2A*, characterized by clustered hotspot variants and relatively higher variant allele fractions compared with other mutations, suggesting that these variants may arise relatively early and potentially provide a selective growth advantage [6]. *UBE2A* encodes an E2 ubiquitin-conjugating enzyme that is involved in the ubiquitin-proteasome system [6]. Given its role in regulated protein degradation, DNA damage response and maintenance of genomic integrity, disruption of this pathway may have functional consequences in endometrial tissue [6]. *UBE2A* alterations may therefore represent a potential early epithelial or mixed-compartment event, but their role in polyp initiation remains insufficiently defined. At present, they should be considered biologically interesting but preliminary findings that require replication and functional validation.

**Figure 1:**
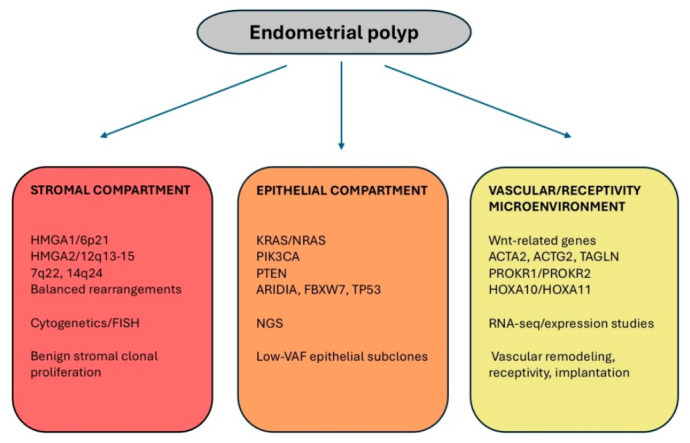
Compartment-specific model of genetic and molecular alterations in endometrial polyps. Stromal-predominant alterations include recurrent balanced chromosomal rearrangements involving *HMGA1/HMGA2* and related loci, mainly detected by cytogenetic and FISH-based approaches. In contrast, sequencing-based studies have identified low-variant-allele-frequency somatic mutations in cancer-associated genes mainly within the epithelial compartment. Gene expression changes affecting Wnt signaling, vascular remodeling, and receptivity-related pathways may contribute to abnormal uterine bleeding and infertility. These alterations may represent distinct but potentially coexisting biological layers within the same lesion.

Sahoo et al. conducted a genomic analysis of 31 benign endometrial polyps and identified 46 somatic mutations across genes such as *PTEN*, *KRAS*, *PIK3CA*, *ARID1A*, *FBXW7* and *TP53* [10]. Importantly, these mutations mirrored the mutational spectrum observed in endometrial carcinoma yet were detected at variant allele frequencies typically below 5%, indicating their presence in only small subclonal populations [10]. It was found that these alterations were confined to the epithelial compartment of the polyps, with little to no involvement of the stromal component. However, Bullerdiek et al. emphasized that stromal chromosomal rearrangements and epithelial somatic mutations in endometrial polyps are not mutually exclusive but rather represent compartment-specific genetic processes that may coexist within the same lesion [3]. Contrary to previous reports, no recurrent gene fusions, copy-number alterations or *HMGA1*/*HMGA2* rearrangements were detected, suggesting that somatic mutations in cancer-associated genes represent secondary events rather than primary drivers of polyp formation [10] (Figure 1). These results support a model in which endometrial polyps serve as long-lived epithelial niches that permit the gradual accumulation of oncogenic mutations without conferring overt malignant behavior [10]. This compartment-specific model helps reconcile the apparent discrepancy between cytogenetic/FISH and NGS studies. Cytogenetic and FISH approaches preferentially identify stromal structural rearrangements, whereas sequencing studies more readily detect low-frequency epithelial mutations. Consequently, these approaches may be capturing different biological layers of the same lesion rather than mutually exclusive pathogenic mechanisms.

In contrast to studies identifying pathogenic or risk-associated factors in endometrial polyps, another study examined common germline polymorphisms in genes involved in estrogen metabolism and receptor signaling and found no association between these polymorphisms and endometrial polyps [16]. In particular, polymorphisms in Catechol O-methylotransferase 2 (*COMT2*), Catechol O-methylotransferase 3 (*COMT3*), Cytochrome P450 1B1 (*CYP1B1*), and Estrogen Receptor 1 (*ESR1*) genes were analyzed and there were no significant differences in allele or genotype frequencies between cases and controls, nor were circulating estradiol and estrone levels affected by these variants [16]. The research implies that polymorphisms in estrogen-related genes do not independently increase the susceptibility to endometrial polyp formation [16]. These negative findings are important because they indicate that not all hormone-related candidate genes contribute measurably to polyp susceptibility. They also highlight the need to distinguish biologically plausible pathways from associations that have been empirically validated.

Moreover, the association between inherited genetic polymorphisms in the insulin-like growth factor (*IGF*) pathway and the risk of developing endometrial polyps was examined by a case–control genetic association analysis [14]. It was revealed that variation in a CA repeat microsatellite within *IGF1* was significantly linked to increased susceptibility to endometrial polyps. Specifically, homozygotes for the *IGF1 CA*(*19*)/*CA*(*19*) genotype, as well as genotypes containing at least one CA repeat longer than 19, showed an increased risk of polyp development [14]. In contrast, a single-nucleotide polymorphism in the IGF-binding protein 3 gene (*IGFBP3*, rs2854746 CG genotype) showed a protective effect, with reduced odds of developing endometrial polyps [14]. Furthermore, concurrent presence of high-risk *IGF1* variants and *IGFBP3* genotypes associated with lower IGF binding capacity was linked to the highest susceptibility to endometrial polyps [14]. In conclusion, this study suggests that germline variation in IGF signaling may influence susceptibility to benign endometrial overgrowth, while complementing broader GWAS data [14]. The IGF-axis findings connect endometrial polyp formation with growth-factor signaling and benign tissue overgrowth. Nevertheless, because these data derive from genetic association analyses, they should be interpreted as risk-modifying rather than diagnostic or mechanistically definitive.

A hospital-based genetic association cohort study examined whether polymorphisms in *LIN28B* affect the recurrence of endometrial polyps after hysteroscopic polypectomy [15]. *LIN28B* encodes an RNA-binding protein which regulates the *LIN28B*/let-7 microRNAs axis, a pathway known to be involved in cellular growth and hyperplastic processes [15]. Specifically, the *LIN28B* rs369065 TT genotype was associated with higher risk of polyp recurrence in comparison to rs314280 A>G which was not associated. Women with single or smaller polyps, that were considered at lower risk of postoperative recurrence, showed an increased risk of recurrence because of the rs369065 polymorphism [15]. These outcomes extend the mutational landscape of endometrial polyps and suggest that germline modulation of proliferation signaling may contribute to polyp persistence and regrowth influencing the clinical behavior [15]. Among the germline findings, *LIN28B* is clinically relevant because it may relate to recurrence after polypectomy rather than initial polyp formation alone. However, its value as a recurrence biomarker remains preliminary until confirmed in independent prospective cohorts.

## 4. Gene Expression Changes and Affected Pathways

**Figure 2:**
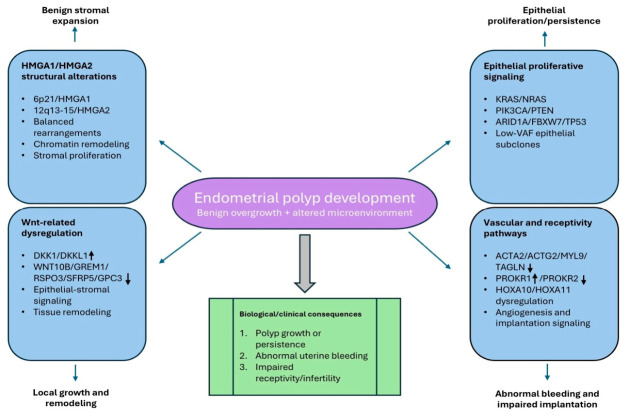
Proposed pathway interaction model in endometrial polyp development. Structural alterations involving *HMGA1/HMGA2* may promote chromatin remodeling and benign stromal proliferation, whereas epithelial proliferative signaling involving *KRAS/NRAS*, *PIK3CA/PTEN*, and other cancer-associated genes may contribute to low-frequency epithelial subclones. Dysregulation of Wnt-related genes may affect epithelial-stromal communication and tissue remodeling, while altered vascular and receptivity-related pathways, including *PROKR1/PROKR2* and *HOXA10/HOXA11*, may contribute to abnormal uterine bleeding and impaired implantation (↑ and ↓ arrows show increased and decreased protein expression, all the other arrows show association with endometrial development). Together, these pathways suggest that endometrial polyps arise through interacting stromal, epithelial, vascular, and receptivity-related mechanisms rather than through a single molecular driver.

The dysregulation of multiple genes and signaling pathways involved in stromal remodeling, vascular regulation and endometrial receptivity are strongly associated with endometrial polyps (Figure 2).

An RNA-seq analysis detected 322 differentially expressed genes (DEGs) between endometrial polyps and adjacent endometrium. Of these, 88 were upregulated and 234 were downregulated in polyps [17]. One of the most significantly altered pathways in polyps is the Wnt signaling pathway [17]. The Wnt signaling pathway is a complex network of protein interactions that plays a key role in embryonic development and cancer, while also contributing to a variety of normal physiological functions in adults [29]. In the context of endometrial polyps, Wnt pathway dysregulation is relevant because it may influence local epithelial-stromal communication, proliferation, and tissue remodeling. Therefore, these expression changes should be interpreted primarily as evidence of altered polyp microenvironment rather than as isolated gene-level abnormalities. Protein—protein interaction and pathway analyses based on differentially expressed genes showed that Dickkopf-1 (*DKK1*) and Dickkopf-like 1 (*DKKL1*) were upregulated while Wnt Family Member 10B (*WNT10B*), Gremlin 1 (*GREM1*), R-spondin 3 (*RSPO3*), Secreted Frizzled-Related Protein 5 (*SFRP5*), and Glypican 3 (*GPC3*) were downregulated. These findings indicate abnormal regulation of growth-controlling pathways that normally restrict endometrial proliferation [17]. Additionally, it was found that genes involved in vascular smooth muscle contraction and cytoskeletal organization including Actin Alpha 2 (*ACTA2*), Actin Gamma 2 (*ACTG2*), Potassium Calcium-Activated Channel Subfamily M Regulatory Beta Subunit 1 (*KCNMB1*), Potassium Calcium-Activated Channel Subfamily M Regulatory Beta Subunit 2 (*KCNMB2*), Myosin Light Chain 9 (*MYL9*), Protein Phosphatase 1 Regulatory Subunit 12B (*PPP1R12B*) and Transgelin (*TAGLN*) were downregulated [17]. These data suggest that defective arteriogenesis and impaired stromal architecture may contribute to abnormal uterine bleeding and impaired embryo implantation in patients with endometrial polyps [17]. Additionally, Gene Ontology analyses further demonstrated disruptions in pathways that are crucial for stromal remodeling and vascular stability such as mesenchymal migration, actin filament organization, focal adhesion, and integrin-mediated signaling [17]. These transcriptomic alterations provide a mechanistic link between molecular dysregulation and the clinical manifestations of endometrial polyps. In particular, impaired vascular smooth muscle function and stromal architecture may contribute to abnormal uterine bleeding, while altered stromal remodeling may disturb the endometrial environment required for implantation.

Beyond Wnt pathway regulation, endometrial polyps have also been shown to alter the expression of genes involved in endometrial receptivity and vascular signaling during the process of implantation. In a comparative gene expression study, samples from women with endometrial polyps were taken during the mid-luteal phase. These samples demonstrated a significant imbalance in the prokineticin pathway [2], a signaling pathway that comprises two secreted proteins, Prokineticin 1 (*PROK-1*) and Prokineticin 2 (*PROK-2*), and two cognate G-protein coupled receptors, Prokineticin Receptor 1 (*PROKR-1*) and Prokineticin Receptor 2 (*PROKR-2*) [30]. While prokineticins were originally known to play an important role in gut motility in the digestive system, it was later found that among other functions they promote angiogenesis in steroidogenic glands, heart and reproductive organs [30]. The results *of* the study indicated increased expression of *PROKR1* and reduced expression of *PROKR2* in comparison to healthy controls, while no significant changes were found in *HOXA10*, *PROK1*, or *PROK2* expression [2]. Homeobox A10 (*HOXA10*) is a member of the homeobox gene family and is a key transcriptional regulator required for uterine development, proper endometrial receptivity, embryo implantation and decidualization of stromal cells and the dysregulation of its expression implies an endometrial disorder [31]. So, the absence of changes in the expression of *HOXA10*, *PROK1* and *PROK2* suggests that the polyps may selectively disrupt downstream receptor-mediated signaling rather than globally impair classical receptivity markers [2]. Altered expression of *PROKR1* and *PROKR2* in the presence of endometrial polyps may therefore impair implantation by affecting vascular and stromal signaling, rather than through direct structural genetic changes. This molecular dysregulation may help explain the association between endometrial polyps and infertility, even when polyps are small or asymptomatic [2]. Thus, *PROKR1*/*PROKR2* dysregulation is more directly relevant to the reproductive consequences of endometrial polyps than to their structural initiation. These findings should be presented as mechanistic evidence for impaired receptivity and implantation, rather than as evidence of a primary genetic driver of polyp formation.

In contrast, another study suggested that *HOXA10* and *HOXA11* expression may be reduced in benign endometrial disorders, including polyps [18]. Both genes are involved in endometrial receptivity, decidualization, and reproductive function [18]. This downregulation may adversely affect stromal decidualization, extracellular matrix remodeling and cytokine-mediated signaling, processes that are essential for successful implantation [18]. It is mainly caused by epigenetic mechanisms, such as promoter hypermethylation and histone modifications, rather than underlying genetic alterations [18]. Taken together, these findings support that *HOXA10* and *HOXA11* dysregulation may contribute to the development of endometrial pathologies such as polyps [18]. The relevance of *HOXA10* and *HOXA11* lies mainly in endometrial receptivity and decidualization. Therefore, their dysregulation may help explain infertility associated with endometrial polyps, but it should be distinguished from stromal chromosomal alterations that are more directly implicated in polyp growth.

## 5. Evidence Hierarchy and Compartment-Specific Interpretation

The available evidence on the genetic background of endometrial polyps should be interpreted according to both methodological approach and tissue compartment analyzed [3,10]. Cytogenetic and FISH-based studies have consistently identified recurrent balanced chromosomal rearrangements, particularly involving 6p21/*HMGA1* and 12q13–15/*HMGA2*, supporting the concept that a subset of endometrial polyps represents benign stromal neoplasms driven by structural genomic alterations [4,6]. In contrast, sequencing-based studies have more frequently detected low-allele-frequency somatic mutations in cancer-associated genes, including *KRAS*, *PIK3CA*, *PTEN*, *ARID1A*, *FBXW7*, and *TP53*, mainly within the epithelial compartment [10].

These apparently discrepant findings should not necessarily be considered contradictory [3]. Cytogenetic and FISH approaches are better suited to detecting balanced rearrangements, large structural changes, and intronic or extragenic breakpoints, whereas targeted or exome-based NGS may fail to capture such alterations, especially when breakpoints fall outside coding regions [3,10]. Conversely, sequencing-based studies have been able to identify low-frequency epithelial mutations that are not typically captured by conventional cytogenetic approaches [10]. Therefore, stromal structural rearrangements and epithelial somatic mutations may represent distinct but potentially coexisting compartment-specific processes in endometrial polyp biology [3,10].

Overall, *HMGA1*/*HMGA2* rearrangements currently represent one of the most reproducible molecular findings in endometrial polyps, particularly in stromal-predominant lesions [6]. By contrast, the clinical and biological significance of low-frequency epithelial mutations, germline susceptibility loci, and gene expression changes remains less well established and requires validation in larger, compartment-resolved cohorts [10,12].

## 6. Clinical Implications

Although genetic and molecular studies have improved understanding of endometrial polyp biology, their current clinical utility remains limited [1,6]. At present, the diagnosis of endometrial polyps continues to rely primarily on imaging, hysteroscopic evaluation, and histopathological confirmation rather than molecular testing [1]. Recurrent *HMGA1*/*HMGA2* rearrangements and stromal structural alterations support the benign neoplastic nature of a subset of polyps, but they are not yet used as routine diagnostic biomarkers [3,6].

Regarding risk stratification, the detection of cancer-associated epithelial mutations such as *KRAS*, *PIK3CA*, *PTEN*, *ARID1A*, *FBXW7*, or *TP53* should be interpreted cautiously [10,11]. These mutations are often present at low variant allele frequencies and may reflect small epithelial subclones rather than malignant transformation [10]. Therefore, their presence alone does not currently justify a change in clinical management [10]. Future studies should determine whether specific molecular profiles are associated with recurrence, persistence, abnormal uterine bleeding, or malignant potential [10,15]. Inflammatory conditions, particularly chronic endometritis, may also be clinically relevant to recurrence risk and should be considered in future risk stratification models [32].

The strongest potential clinical relevance lies in reproductive medicine [1,2]. Altered expression of genes involved in endometrial receptivity, vascular regulation, and implantation-related signaling, including *PROKR1*/*PROKR2* and *HOXA10*/*HOXA11*, may help explain the association between endometrial polyps and infertility [2,18]. However, these findings remain mainly mechanistic and should not yet be considered validated biomarkers of implantation failure [2,18]. Larger prospective studies integrating molecular findings with fertility outcomes after polypectomy are needed before these markers can be incorporated into reproductive counseling or personalized management [2,15].

## 7. Conclusions

This review highlights the multifactorial genetic landscape underlying the development of endometrial polyps. After collecting evidence from multiple studies, available evidence suggests that these lesions cannot be explained by a single pathogenic mechanism, but rather arise through the interaction of chromosomal alterations, somatic and germline genetic variants, and dysregulated gene expression. Current evidence supports the interpretation that a substantial subset of endometrial polyps represents benign stromal neoplasms driven by balanced genetic alterations, involving genes that play a role in pathways related to stromal remodeling, vascular stability, endometrial receptivity and implantation or genes that are associated with endometrial carcinogenesis [33]. Overall, endometrial polyps can be considered as genetically heterogeneous lesions arising from compartment-specific alterations, with stromal structural changes and epithelial mutational events coexisting within the same lesion [32]. Future studies integrating genome-wide structural, mutational and epigenetic analyses in large, well-defined cohorts will be necessary in order to further identify clinically relevant biomarkers that could improve risk stratification, reproductive counseling and personalized management of women with endometrial polyps.

## 8. Future Directions

Despite the high prevalence of endometrial polyps, the overall genetic background of these lesions remains incompletely understood. It is suggested that endometrial polyps arise from a multifactorial genetic background involving both structural genomic alterations and molecular signaling dysregulation, yet research in this field is still in its infancy. Gaps in research appear due to the apparent inconsistency between cytogenetic studies that identify recurrent, clonal structural rearrangements (especially involving the *HMGA* loci) and sequencing-based studies that have been unable to reproduce these results as a constant feature, highlighting the need for harmonized detection methods and case selection criteria [3,10]. Studies separating stromal and epithelial compartments are essential because epithelial driver mutations frequently present at low allelic fractions, while stromal alterations appear to act as primary structural drivers, and current studies differ substantially in the extent to which epithelial and stromal compartments are independently analyzed [6,10]. Although genome-wide analyses increasingly support a model characterized by balanced structural rearrangements with minimal copy-number alterations, further studies are required to define the functional roles of recurrent partner loci, such as 14q24, and to determine whether these define consistent molecular endotypes across clinical settings [6,24]. Future studies should extend beyond the identification of rearrangements and mutations to directly test their downstream transcriptional consequences, using evidence of stromal overexpression signatures, including *HMGA*-driven transcriptional activation, and integrating these findings with pathway-level transcriptomic analyses [6,17]. Prospective longitudinal cohorts are essential in order to link molecular subtypes with clinical outcomes such as recurrence, as germline variants like *LIN28B* are rarely integrated with somatic and structural profiling in the same patients [6,15]. In addition, germline risk variants identified by large Biobank GWAS require replication, fine-mapping, and mechanistic validation, as smaller studies report either positive associations (*IGF*-axis variants) or null findings (*COMT*, *CYP1B1* and *ESR1* polymorphisms), which underscores heterogeneity across populations and study designs [12,14,16]. Initiating somatic alterations must be distinguished from secondary mutations that accumulate with lesion persistence, a distinction that will require standardized ultra-deep sequencing approaches [6,10,11]. Future studies should investigate whether higher-allelic-fraction, hotspot-like mutations, such as *UBE2A*, provide a growth advantage and whether they have clinical significance [6]. Future studies should focus on the clarification of the relationship between specific molecular alterations and implantation failure, as the dysregulation of some genes (prokineticin receptor imbalance, *HOXA10*/*HOXA11*) has been associated with impaired endometrial receptivity [2]. In addition, non-genetic factors implicated in endometrial polyp formation, such as altered progesterone responsiveness and localized chronic inflammation, require further molecular investigation [1]. Although these mechanisms have been proposed to contribute to persistent endometrial proliferation, tissue remodeling, angiogenesis, and impaired cyclic regression, direct studies linking progesterone-related pathways or inflammatory mediators to specific genetic or transcriptomic subtypes of endometrial polyps remain limited. Recent clinical evidence also suggests that chronic endometritis may increase the recurrence risk of endometrial polyps after transcervical resection, supporting the need to integrate inflammatory markers with molecular profiling in future studies. Future research should therefore examine whether progesterone signaling and local inflammatory pathways interact with stromal chromosomal rearrangements, epithelial somatic mutations, or receptivity-related gene expression changes during polyp development. Resolving the marked heterogeneity of endometrial polyps will require integrated multi-omic approaches that combine cytogenetic or whole-genome sequencing data with deep mutational and transcriptomic profiling in the same lesions, paired with clinical metadata, in order to facilitate the development of clinically useful biomarkers and management strategies [6,15,27].

## Figures and Tables

**Table 1:** Molecular alterations in endometrial polyps.

Chromosome/Gene Affected	Tissue Compartment/Biological Level	Molecular Category	Evidence Type	Sample Size/Study Scale	Main Finding	Biological Relevance to Endometrial Polyps	Associated Disease/Clinical Context	Key References
6p21/*HMGA1*	Stroma/mesenchymal compartment	Chromosomal structural alterations	Cytogenetics, FISH, genomic profiling	33 polyps in classic cytogenetic study; additional case reports/series; genomic profiling cohort	Recurrent rearrangements involving 6p21/*HMGA1*	Supports benign stromal clonal proliferation and the mesenchymal/stromal neoplastic nature of a subset of polyps	Similar structural alterations reported in benign mesenchymal tumors such as leiomyomas and lipomas; not currently used as a clinical biomarker	[4,5,6]
*PLAG1*	Stroma/mesenchymal compartment	Downstream transcriptional activation	Gene expression/downstream pathway interpretation	23 polyps in genomic profiling and expression study	Suggested downstream upregulation associated with *HMGA1* alterations	May contribute to proliferation-related transcriptional programs in stromal cells	Mechanistic relevance; no established diagnostic use	[6]
12q13–15/*HMGA2*	Stroma/mesenchymal compartment	Chromosomal structural alterations	Cytogenetics, FISH, IHC, genomic profiling	Case reports/series; 23 polyps plus 54 validation samples in genomic profiling study	Rearrangement, amplification, or overexpression of *HMGA2*	Promotes benign stromal overgrowth and clonal expansion through chromatin-related transcriptional regulation	Shared context with benign mesenchymal tumors, including uterine leiomyomas and lipomas; not a routine diagnostic biomarker	[6,7,8]
7q22	Stroma/mesenchymal compartment	Chromosomal structural alterations	Cytogenetics	33 polyps in classic cytogenetic study; 7q22 subgroup reported in 2/33 polyps	Rare rearrangements involving 7q22	Biological significance remains insufficiently defined; should be considered preliminary	No established clinical or disease-specific application	[4]
14q24	Stroma/mesenchymal compartment	Chromosomal structural alterations	Cytogenetics, genomic profiling	3 stromal-predominant lesions in cytogenetic study; 23 polyps plus 54 validation samples in genomic profiling study	Reported as a partner region in balanced rearrangements, including t(6;14)(p21;q24)	Expands the spectrum of structural alterations in stromal-predominant polyps	Preliminary molecular subgroup; no established clinical use	[6,9]
*KRAS*	Epithelium/epithelial compartment	Somatic mutations	WES/targeted sequencing	4 polyps initially analyzed by WES and 35 polyps by targeted mutation analysis; 31 benign polyps in the NGS study	Activating KRAS mutations reported in benign polyps	May promote epithelial proliferation and contribute to multiple polyp formation; should not alone imply malignancy	Cancer-associated gene, but in benign polyps usually interpreted cautiously	[10,11]
*NRAS*	Epithelium/epithelial compartment	Somatic mutations	WES/targeted sequencing	35 polyps in targeted mutation analysis	Less frequent RAS-family mutation compared with KRAS	May contribute to epithelial proliferative signaling in a subset of lesions	Cancer-associated signaling pathway; clinical significance in benign polyps remains unproven	[11]
*PIK3CA*	Epithelium/epithelial compartment	Somatic mutations	NGS	31 benign polyps in NGS study; also assessed in 23 polyps plus 54 validation samples in genomic profiling study	Low-allele-frequency epithelial mutation	Suggests small epithelial subclones within otherwise benign lesions	Endometrial carcinoma-associated gene, but not sufficient alone for malignant risk stratification	[10]
*PTEN*	Epithelium/epithelial compartment	Somatic mutations	NGS	31 benign polyps in NGS study; also assessed in 23 polyps plus 54 validation samples in genomic profiling study	Low-allele-frequency epithelial mutation	May reflect accumulation of epithelial mutations in long-lived benign lesions	Endometrial carcinoma-associated gene; clinical significance remains uncertain	[10]
*ARID1A*	Epithelium/epithelial compartment	Somatic mutations	NGS	31 benign polyps in NGS study	Low-allele-frequency epithelial mutation	Indicates that cancer-associated mutations may occur in benign epithelial subclones	Cancer-associated chromatin remodeling gene; not currently a stand-alone clinical marker in polyps	[10]
*FBXW7*	Epithelium/epithelial compartment	Somatic mutations	NGS	31 benign polyps in NGS study	Low-allele-frequency epithelial mutation	May represent a secondary epithelial event rather than a primary polyp driver	Cancer-associated gene; clinical significance in benign polyps remains unclear	[10]
*TP53*	Epithelium/epithelial compartment	Somatic mutations	NGS	31 benign polyps in NGS study	Low-allele-frequency epithelial mutation	Should be interpreted cautiously in a benign histological context	Strongly cancer-associated gene, but low-VAF detection in benign polyps does not by itself indicate malignant transformation	[10]
*UBE2A*	Epithelium/possible mixed compartment	Somatic mutations	Genomic profiling/sequencing	23 polyps plus 54 validation samples in genomic profiling study	Hotspot-like variants with relatively higher variant allele fractions were reported in a subset	May represent an early mutation or growth-advantage event, but functional role remains preliminary	Candidate marker requiring validation; no established clinical use	[6]
*EXO1*	Germline/inherited susceptibility	Germline susceptibility loci	GWAS/variant-to-gene prioritization	36,984 women with female genital tract polyps and 420,993 controls	Susceptibility locus linked to DNA repair pathways	Suggests an inherited contribution to genomic stability and benign tissue overgrowth	Associated with DNA damage repair biology; not used for individual risk prediction	[12]
*CHEK2*	Germline/inherited susceptibility	Germline susceptibility loci	GWAS/variant-to-gene prioritization	36,984 women with female genital tract polyps and 420,993 controls	Susceptibility locus related to checkpoint control and DNA damage response	Links polyp susceptibility to cell-cycle and DNA repair regulation	Cancer predisposition-related pathway, but clinical relevance in polyps remains investigational	[12]
*PRIM1*	Germline/inherited susceptibility	Germline susceptibility loci	GWAS/variant-to-gene prioritization	36,984 women with female genital tract polyps and 420,993 controls	Susceptibility locus related to DNA replication	May contribute to inherited predisposition through replication/proliferation pathways	Risk-modifying candidate; not diagnostic	[12]
*PSMD13*	Germline/inherited susceptibility	Germline susceptibility loci	GWAS/variant-to-gene prioritization	36,984 women with female genital tract polyps and 420,993 controls	Susceptibility locus linked to proteasome-related regulation	May influence protein turnover, cell-cycle control, and benign overgrowth	Investigational susceptibility gene	[12]
*MYNN*	Germline/inherited susceptibility	Germline susceptibility loci	GWAS/variant-to-gene prioritization	36,984 women with female genital tract polyps and 420,993 controls	Susceptibility locus overlapping with regions implicated in other gynecologic conditions	May contribute to proliferative susceptibility and shared genetic architecture	Reported overlap with uterine fibroids/endometriosis-related genetic architecture	[12]
*LRRC34*	Germline/inherited susceptibility	Germline susceptibility loci	GWAS/variant-to-gene prioritization	36,984 women with female genital tract polyps and 420,993 controls	Susceptibility locus reported among prioritized genes	May indicate an inherited predisposition involving proliferative or repair-related pathways	Reported overlap with uterine fibroids/endometriosis-related genetic architecture	[12]
*ODF3*	Germline/inherited susceptibility	Germline susceptibility loci	GWAS/variant-to-gene prioritization	36,984 women with female genital tract polyps and 420,993 controls	Susceptibility locus prioritized in GWAS	Potential contribution to inherited susceptibility; precise mechanism in polyps remains uncertain	Investigational; not clinically used	[12]
*POL3*	Germline/inherited susceptibility	Germline susceptibility loci	GWAS/variant-to-gene prioritization	36,984 women with female genital tract polyps and 420,993 controls	Gene related to DNA polymerase function mentioned among DNA repair/replication candidates	May link polyp susceptibility to DNA replication fidelity	Investigational susceptibility signal	[12,13]
*SFR1*	Germline/inherited susceptibility	Germline susceptibility loci	GWAS/variant-to-gene prioritization	36,984 women with female genital tract polyps and 420,993 controls	Candidate gene related to cellular proliferation pathways	May contribute to inherited susceptibility through proliferative regulation	Investigational; no direct clinical use	[12]
*PLCE1*	Germline/inherited susceptibility	Germline susceptibility loci	GWAS/variant-to-gene prioritization	36,984 women with female genital tract polyps and 420,993 controls	Candidate gene related to signaling/proliferation	May contribute to benign tissue overgrowth susceptibility	Investigational risk locus	[12]
*ZBTB38*	Germline/inherited susceptibility	Germline susceptibility loci	GWAS/variant-to-gene prioritization	36,984 women with female genital tract polyps and 420,993 controls	Candidate susceptibility gene	May relate to growth regulation or cellular proliferation	Investigational; not used clinically	[12]
*NFIA*	Germline/inherited susceptibility	Germline susceptibility loci	GWAS/variant-to-gene prioritization	36,984 women with female genital tract polyps and 420,993 controls	Candidate susceptibility gene	May contribute to transcriptional or developmental regulation relevant to tissue growth	Investigational; no established clinical application	[12]
*EEFSEC*	Germline/inherited susceptibility	Germline susceptibility loci	GWAS/variant-to-gene prioritization	36,984 women with female genital tract polyps and 420,993 controls	Candidate locus overlapping with other gynecologic traits	Suggests shared genetic architecture with benign gynecologic conditions	Reported in relation to uterine fibroids/endometriosis-associated loci	[12]
*IGF1*	Germline/growth-factor susceptibility	Germline association	Case–control genetic association analysis	104 women with a history of endometrial polyp and 81 postmenopausal controls	IGF1CA-repeat variants associated with increased polyp susceptibility	Links growth-factor signaling to benign endometrial overgrowth	Potential risk-modifying pathway; not used for clinical genotyping	[14]
*IGFBP3*	Germline/growth-factor susceptibility	Germline association	Case–control genetic association analysis	104 women with a history of endometrial polyp and 81 postmenopausal controls	IGFBP3 variant reported as potentially protective	May modify IGF bioavailability and growth signaling	Risk-modifying candidate; no routine clinical use	[14]
*LIN28B*	Germline/recurrence risk	Germline polymorphism	Hospital-based genetic association cohort	351 reproductive-age women with endometrial polyps after hysteroscopic polypectomy	rs369065TT genotype associated with increased postoperative recurrence risk	May influence recurrence through LIN28B/let-7growth-regulatory axis	Candidate recurrence marker after polypectomy; requires validation	[15]
*COMT2*	Hormone-related candidate genes	Germline polymorphisms/negative association study	Candidate gene association study	309 women total: 236 with endometrial polyps and 73 controls without hysteroscopic abnormalities	No significant association with endometrial polyps	Suggests not all estrogen metabolism genes contribute measurably to polyp susceptibility	Hormone metabolism pathway; negative/inconclusive clinical relevance	[16]
*COMT3*	Hormone-related candidate genes	Germline polymorphisms/negative association study	Candidate gene association study	309 women total: 236 with endometrial polyps and 73 controls without hysteroscopic abnormalities	No significant association with endometrial polyps	Indicates limited evidence for this estrogen metabolism variant in polyp formation	Hormone metabolism pathway; not clinically useful	[16]
*CYP1B1*	Hormone-related candidate genes	Germline polymorphisms/negative association study	Candidate gene association study	309 women total: 236 with endometrial polyps and 73 controls without hysteroscopic abnormalities	No significant association with endometrial polyps	Does not support a clear independent role for this estrogen metabolism gene	Estrogen metabolism pathway; no established clinical use	[16]
*ESR1*	Hormone-related candidate genes	Germline polymorphisms/negative association study	Candidate gene association study	309 women total: 236 with endometrial polyps and 73 controls without hysteroscopic abnormalities	No significant association with endometrial polyps	Does not support a clear independent role for this estrogen receptor gene in polyp susceptibility	Hormone receptor pathway; not clinically useful in current practice	[16]
*DKK1*	Mixed tissue/epithelial-stromal signaling	Gene expression/Wnt signaling	RNA-seq/differential gene expression	12 paired endometrial polyp and adjacent endometrial tissue samples	Upregulated in endometrial polyps	Suggests altered Wnt pathway regulation and local proliferation/remodeling	Mechanistic relevance; not a diagnostic biomarker	[17]
*DKKL1*	Mixed tissue/epithelial-stromal signaling	Gene expression/Wnt signaling	RNA-seq/differential gene expression	12 paired endometrial polyp and adjacent endometrial tissue samples	Upregulated in endometrial polyps	May reflect dysregulated Wnt-related signaling in the polyp microenvironment	Mechanistic relevance; no established clinical use	[17]
*WNT10B*	Mixed tissue/epithelial-stromal signaling	Gene expression/Wnt signaling	RNA-seq/differential gene expression	12 paired endometrial polyp and adjacent endometrial tissue samples	Downregulated in endometrial polyps	Suggests disrupted Wnt-mediated epithelial-stromal communication	Mechanistic relevance; not used clinically	[17]
*GREM1*	Mixed tissue/epithelial-stromal signaling	Gene expression/Wnt-related regulation	RNA-seq/differential gene expression	12 paired endometrial polyp and adjacent endometrial tissue samples	Downregulated in endometrial polyps	May contribute to altered growth-control and stromal remodeling pathways	Mechanistic relevance; no routine clinical application	[17]
*RSPO3*	Mixed tissue/epithelial-stromal signaling	Gene expression/Wnt-related regulation	RNA-seq/differential gene expression	12 paired endometrial polyp and adjacent endometrial tissue samples	Downregulated in endometrial polyps	Suggests altered Wnt modulation and tissue remodeling	Mechanistic relevance	[17]
*SFRP5*	Mixed tissue/epithelial-stromal signaling	Gene expression/Wnt antagonist/modulator	RNA-seq/differential gene expression	12 paired endometrial polyp and adjacent endometrial tissue samples	Downregulated in endometrial polyps	May reflect disruption of local Wnt pathway balance	Mechanistic relevance	[17]
*GPC3*	Mixed tissue/epithelial-stromal signaling	Gene expression/Wnt-related regulation	RNA-seq/differential gene expression	12 paired endometrial polyp and adjacent endometrial tissue samples	Downregulated in endometrial polyps	May contribute to altered growth factor/Wnt-related microenvironmental signaling	Mechanistic relevance; no established clinical use	[17]
*ACTA2*	Vasculature/stromal-vascular compartment	Cytoskeletal and vascular gene expression	RNA-seq/differential gene expression	12 paired endometrial polyp and adjacent endometrial tissue samples	Downregulated in endometrial polyps	Suggests impaired vascular smooth muscle function and stromal architecture	May relate mechanistically to abnormal uterine bleeding	[17]
*ACTG2*	Vasculature/stromal-vascular compartment	Cytoskeletal and vascular gene expression	RNA-seq/differential gene expression	12 paired endometrial polyp and adjacent endometrial tissue samples	Downregulated in endometrial polyps	Supports altered contractile/cytoskeletal organization	Potential relevance to bleeding and stromal remodeling	[17]
*KCNMB1*	Vasculature/stromal-vascular compartment	Vascular smooth muscle signaling	RNA-seq/differential gene expression	12 paired endometrial polyp and adjacent endometrial tissue samples	Downregulated in endometrial polyps	May indicate altered vascular tone and smooth muscle function	Potential mechanistic link to abnormal uterine bleeding	[17]
*KCNMB2*	Vasculature/stromal-vascular compartment	Vascular smooth muscle signaling	RNA-seq/differential gene expression	12 paired endometrial polyp and adjacent endometrial tissue samples	Downregulated in endometrial polyps	May contribute to impaired vascular regulation	Potential relevance to bleeding symptoms	[17]
*MYL9*	Vasculature/stromal-vascular compartment	Cytoskeletal and contractile gene expression	RNA-seq/differential gene expression	12 paired endometrial polyp and adjacent endometrial tissue samples	Downregulated in endometrial polyps	Suggests altered actomyosin contractility and stromal organization	Mechanistic link to vascular/stromal dysfunction	[17]
*PPP1R12B*	Vasculature/stromal-vascular compartment	Cytoskeletal and contractile gene expression	RNA-seq/differential gene expression	12 paired endometrial polyp and adjacent endometrial tissue samples	Downregulated in endometrial polyps	May contribute to altered smooth muscle contraction and vascular stability	Potential relevance to abnormal bleeding; not clinically validated	[17]
*TAGLN*	Vasculature/stromal-vascular compartment	Cytoskeletal and stromal remodeling	RNA-seq/differential gene expression	12 paired endometrial polyp and adjacent endometrial tissue samples	Downregulated in endometrial polyps	Suggests impaired cytoskeletal organization and stromal remodeling	Potential relevance to bleeding and implantation environment	[17]
*PROK1*	Receptivity/implantation-related endometrium	Prokineticin signaling	Gene expression study	15 endometrial polyp patients, 21 myoma uteri patients, and 23 healthy controls	No significant expression change was reported in one study	Suggests that polyps may selectively affect receptor-mediated signaling rather than ligand expression	Reproductive/implantation context; no clinical biomarker role	[2]
*PROK2*	Receptivity/implantation-related endometrium	Prokineticin signaling	Gene expression study	15 endometrial polyp patients, 21 myoma uteri patients, and 23 healthy controls	No significant expression change was reported in one study	Suggests selective disruption of downstream receptor signaling rather than global prokineticin pathway change	Reproductive/implantation context	[2]
*PROKR1*	Receptivity/implantation-related endometrium	Prokineticin receptor signaling	Gene expression study	15 endometrial polyp patients, 21 myoma uteri patients, and 23 healthy controls	Upregulated in the endometrium from women with polyps	May affect vascular and stromal signaling involved in implantation	Potential mechanistic link to infertility; not a validated fertility biomarker	[2]
*PROKR2*	Receptivity/implantation-related endometrium	Prokineticin receptor signaling	Gene expression study	15 endometrial polyp patients, 21 myoma uteri patients, and 23 healthy controls	Downregulated in the endometrium from women with polyps	May disrupt receptivity-related signaling and implantation environment	Potential mechanistic link to infertility; not clinically validated	[2]
*HOXA10*	Receptivity/decidualization-related endometrium	*HOX* gene expression/epigenetic regulation	Gene expression/epigenetic studies	15 endometrial polyp patients, 21 myoma uteri patients, and 23 healthy controls in a gene expression study; review-level evidence in benign endometrial disorders	Reported as unchanged in one study and reduced/dysregulated in another context	Important for endometrial receptivity, decidualization, and implantation	Infertility/receptivity relevance; not a routine biomarker for polypectomy decisions	[2,18]
*HOXA11*	Receptivity/decidualization-related endometrium	*HOX* gene expression/epigenetic regulation	Gene expression/epigenetic studies	Review article; no original endometrial polyp sample size	Reported as reduced/dysregulated in benign endometrial disorders including polyps	May impair decidualization, extracellular matrix remodeling, and implantation	Infertility/receptivity relevance; not clinically validated as predictive biomarker	[18]

## Data Availability

No new data were created or analyzed in this study. Data sharing is not applicable to this article.

## Abbreviations

The following abbreviations are used in this manuscript:

SNP	Single Nucleotide Polymorphism
*HMGA1*	High Mobility Group AT-hook 1
*PLAG1*	Pleomorphic Adenoma Gene 1
*HMGA2*	High Mobility Group AT-hook 2
*FISH*	Fluorescence In Situ Hybridization
*V2G*	Variant-to-Gene
*ODF3*	Outer Dense Fiber of Sperm Tails 3
*PSMD13*	26S Proteasome Non-ATPase Regulatory Subunit 13
*POL3*	DNA Polymerase Delta Catalytic Subunit
*LRRC34*	Leucine Rich Repeat Containing 34
*MYNN*	Myoneurin Gene
*EXO1*	Exonuclease 1
*CHEK2*	Checkpoint Kinase 2
*PRIM1*	DNA Primase Subunit 1
*SFR1*	Swi5-Sfr1 Homolog
*PLCE1*	Phospholipase C Epsilon 1
*ZBTB38*	Zinc Finger and BTB Domain Containing 38
*NFIA*	Nuclear Factor I A
*EEFSEC*	Eukaryotic Elongation Factor, Selenocysteine-Specific
*BMI*	Body Mass Index
*SHBG*	Sex Hormone-Binding Globulin
*KRAS*	Kirsten Rat Sarcoma Viral Oncogene Homolog
*NRAS*	Neuroblastoma RAS Viral Oncogene Homolog
*UBE2A*	Ubiquitin-Conjugating Enzyme E2 A
*PTEN*	Phosphatase and Tensin Homolog
*PIK3CA*	Phosphatidylinositol-4,5-Bisphosphate 3-Kinase Catalytic Subunit Alpha
*ARID1A*	AT-Rich Interaction Domain 1A
*FBXW7*	F-Box and WD Repeat Domain Containing 7
*TP53*	Tumor Protein p53
*COMT2*	Catechol-O-Methyltransferase 2
*COMT3*	Catechol-O-Methyltransferase 3
*CYP1B1*	Cytochrome P450 Family 1 Subfamily B Member 1
*ESR1*	Estrogen Receptor 1
*IGF1*	Insulin-Like Growth Factor 1
*IGFBP3*	Insulin-Like Growth Factor Binding Protein 3
*LIN28B*	Lin-28 Homolog B
*DEGs*	Differentially Expressed Genes
*DKK1*	Dickkopf-1
*DKKL1*	Dickkopf-Like 1
*WNT10B*	Wnt Family Member 10B
*GREM1*	Gremlin 1
*RSPO3*	R-Spondin 3
*SFRP5*	Secreted Frizzled-Related Protein 5
*GPC3*	Glypican 3
*ACTA2*	Actin Alpha 2
*ACTG2*	Actin Gamma 2
*KCNMB1*	Potassium Calcium-Activated Channel Subfamily M Regulatory Beta Subunit 1
*KCNMB2*	Potassium Calcium-Activated Channel Subfamily M Regulatory Beta Subunit 2
*MYL9*	Myosin Light Chain 9
*PPP1R12B*	Protein Phosphatase 1 Regulatory Subunit 12B
*TAGLN*	Transgelin
*PROK1*	Prokineticin 1
*PROK2*	Prokineticin 2
*PROKR1*	Prokineticin Receptor 1
*PROKR2*	Prokineticin Receptor 2
*HOXA10*	Homeobox A10
*HOXA11*	Homeobox A11
